# *Ecytonucleospora hepatopenaei* (EHP) disease prevalence and mortality in *Litopenaeus vannamei*: a comparative study from Eastern India shrimp farms

**DOI:** 10.1186/s12866-024-03681-y

**Published:** 2024-12-19

**Authors:** Vikash Kumar, Basanta Kumar Das, Souvik Dhar, Kampan Bisai, Gde Sasmita Julyantoro Pande, Xiaoting Zheng, Satya Narayan Parida, Anupam Adhikari, Asim Kumar Jana

**Affiliations:** 1 Aquatic Environmental Biotechnology (AEB) Division, ICAR-Central Inland Fisheries Research Institute (CIFRI), Barrackpore, 700120 India; 2 ICAR-Central Inland Fisheries Research Institute (CIFRI), Barrackpore, 700120 India; 3 Department of Aquatic Resource Management, Faculty of Marine Science and Fisheries, Udayana University, Bali, Kuta Selatan 80361 Indonesia; 4 Key Laboratory of South China Sea Fishery Resources Exploitation & Utilization, Ministry of Agriculture and Rural Affairs, South China Sea Fisheries Research Institute, Chinese Academy of Fishery Sciences, Guangzhou, 510300 China

**Keywords:** EHP, *Litopenaeus vannamei*, Morality, Gene expression, Gut bacteria

## Abstract

*Ecytonucleospora hepatopenaei* (EHP), a microsporidian parasite first named and characterized from the *Penaeus monodon* (black or giant tiger shrimp), causes growth retardation and poses a significant threat to shrimp farming. We observed shrimp farms associated with disease conditions during our fish disease surveillance and health management program in West Bengal, India. Shrimp exhibited growth retardation and increased size variability, particularly in advanced stages, exhibiting soft shells, lethargy, reduced feeding and empty midguts. Floating white feces were observed on the surface of the pond water. Suspecting a microbial infection, the shrimp samples were collected and aseptically brought to the ICAR-CIFRI laboratory for molecular confirmation. A nested PCR was used to screen shrimp tissue, feces, feed and environmental samples for the possible presence of hepatopancreatic microsporidiosis caused by *Ecytonucleospora hepatopenaei*. The results confirmed that the shrimp samples were positive for EHP. Histopathological investigation revealed mature spores in the HP tubule lumen and epithelial cells along with necrotic tubule in the symptomatic group. Further, the transcription analysis revealed that ProPO, Hsp70 and α2-macroglobulin genes were significantly upregulated, while decreased expression of LGBP, PXN and Integrin ß was observed in shrimp infected with Hepatopancreatic microsporidiosis. Furthermore, compared with the healthy group, significant intestinal bacteria changes were observed in the EHP-infected group. The in vivo survival assay, using crustacean animal model *Artemia franciscana*, suggests that symptomatic shrimp gut samples harbour pathogenic *Vibrio parahaemolyticus*, *V. harveyi* and *V. campbellii*. These results significantly advance our understanding of the molecular and ecological aspects of EHP pathobiology.

## Introduction

The shrimp farming industry has a high economic value in many countries worldwide. The high demand in international markets and global consumption resulted in a rapid expansion of the shrimp aquaculture industry, especially the culture of the Pacific white shrimp *Litopenaeus vannamei*, which is now recognized as a principal species for commercial shrimp farming due to its rapid growth rate and high nutritional value [1]. India is the second-largest aquaculture-producing country, contributing around 17.5% of the global farmed shrimp production. However, the major obstacle to successful shrimp farming comes from several disease outbreaks caused by bacterial, viral, and parasitic infections [2, 3]. These infections lead to significant economic losses. Recently, an emerging disease called hepatopancreatic microsporidiosis (HPM) has become a significant concern in the shrimp farming industry [4]. HPM is caused by a microsporidian parasite *Enterocytozoon hepatopenaei* (EHP). Microsporidians are obligate intracellular spore-forming pathogens in some protists, invertebrates and vertebrates. As a ubiquitous pathogen, their spread in freshwater, brackish water and marine environments has been extensively documented [5]. EHP infection is associated with growth retardation and severe size variation, resulting in a reduction in shrimp biomass production [6]. It is suggested that EHP infection could make the shrimp weaken. Hence, they are more susceptible to other diseases.

EHP was first reported to infect the black tiger shrimp *Penaeus monodon* in Thailand in 2004 [6]. Since then, it has been widespread in many Asian countries, including Korea, China, Indonesia, India, Vietnam, and Malaysia. EHP infection resulted in a production loss of 0.77 million tons, reducing revenue by US$ 567.62 million in Indian shrimp farms. The main clinical signs of EHP are growth retardation, which leads to increased size variability [7]. Whitish fecal strings floating on the surface of pond water and the presence of shrimp displaying white discoloration of the gastrointestinal tract (GI tract) in these ponds have also been associated with EHP [8]. In advanced stages of the disease, EHP-infected shrimp typically display soft shells, lethargy, reduced feed intake, an empty midgut, and chronic mortalities. EHP is an intracellular microsporidium that causes lesions in the hepatopancreas (HP) tubule epithelial cells [9, 10]. EHP replicates within the cytoplasm of the affected cells. The histology of EHP-infected shrimp shows irregular/regular basophilic inclusion bodies within the cytoplasm with or without spores [6, 9, 11–13]. Additional histopathological lesions include mild to severe sloughing of the tubular epithelial cells, usually with the presence of mature spores. Moreover, the spores are also observed in the lumen of the HP tubules and the GI tract [14–16].

The hepatopancreas is the main organ that plays a major role in digestive enzyme secretion, food digestion, absorption, and storage of lipids, glycogen, and minerals [17]. The target organ for the microsporidian EHP infection is the shrimp hepatopancreas, causing the shedding of hepatic tubular epithelial cells and atrophy. Although EHP infection does not result in significant mortality, the infection may affect digestive enzyme secretion, nutrient digestion, absorption, and metabolism [15–19]. EHP causes different body sizes and growth retardation of shrimp, while at a late stage of EHP infection, shrimp usually exhibit soft shells, reduced food intake, drowsiness, and other physiological phenomena [20–23]. Relevant studies from the past few decades have overlooked the functions of gut microbiota and immune response in favor of concentrating on identifying EHP and the physiological changes that occur in shrimp following EHP infection [20, 24, 25]. It is generally accepted that gut microbiota plays essential roles, including acquiring nutrients, the pathogen invasion barrier, and immunoregulation in the host [26, 27]. On the contrary, the creation of the gut microbiota could be significantly disrupted by pathogenic invasions [28, 29].

In the present study, the research focuses on determining the etiological agent responsible for growth retardation and size variation of *Litopenaeus vannamei* (Pacific white shrimp) in a shrimp farm in West Bengal, India. The EHP was confirmed as a causative agent associated with disease conditions through microscopy and molecular analysis. Using the crustacean animal model *Artemia franciscana*, we explored how the gut microbiota of shrimp was affected by EHP infection by characterization of isolated gut bacteria virulence. Further, the effects of EHP infection on the histology of hepatopancreas and the immune response of shrimp were investigated. The knowledge gained from the study would facilitate future research to better understand the host-pathogen response during EHP infection.

## Materials and methods

### Shrimp mortality and sample collection

The *Litopenaeus vannamei* (pacific white shrimp) cultured in grow-out ponds in South 24 Parganas and East Midnapore district, West Bengal, India, exhibited disease clinical signs, specifically significant size variation between individual shrimp and reduced growth. Based on the farmers’ reports from early September 2022 to August 2023, the ICAR-CIFRI (a nodal agency of disease surveillance in Eastern India) team of researchers visited the farm facilities. The shrimp, water, and sediment samples collected from 120 representative shrimp farms (9 samples/pond) were used for the analysis. Fresh smears of gills and hepatopancreas were examined under a microscope in the lab to check for ectoparasites. The shrimp were then euthanized by an overdose of MS222 (Sigma-Aldrich), and post-mortem exams were carried out to document various clinical signs in the internal organs. For molecular studies, different tissues from symptomatic and asymptomatic shrimp tissue samples (gut and hepatopancreas) were collected in RNA later. Further, the hepatopancreas were fixed in Davidson’s solution for 48 h and placed in 70% ethanol for histopathological analysis. The post-mortem and clinical examinations were conducted according to established procedures [30].

### PCR assay for detection of *Ecytonucleospora hepatopenaei*

The hepatopancreas of shrimp (average 30 mg) from representative shrimp farms were sampled and pooled for PCR analysis. DNeasy Blood & Tissue kits (Qiagen, India) were used to extract DNA following the manufacturer’s instructions. Additionally, soil and pond water samples from the shrimp ponds were used for DNA extraction. A 1% agarose gel was utilized to evaluate the purity of the extracted DNA, and a nanodrop (Eppendorf, India) was used to measure the quantity. Previous work by Tangprasittipap et al. [7] used two rounds of nested PCR to amplify the small subunit ribosomal RNA (ssu rRNA) gene of EHP. SWP_1F and SWP_1R primers were used in the first PCR reaction for the nested PCR of the SWP gene (spore wall protein), and SWP_2F and SWP_2R primers were used in the second PCR reaction (Table 1). One µL of 50 mM MgCl_2_, one µL of 10 mM dNTP, one µL of 10 pmol of each primer, one U Taq DNA polymerase, and five µL of 10× PCR buffer was added to each PCR mixture to create a final volume of 50 µL. The PCR profile was as follows: 3 min at 94 °C for initial denaturation; 40 cycles of 30 s at 94 °C for denaturation; 60 s at 64 °C for annealing; 60 s at 72 °C for extension; and a final extension lasting three minutes at 72 °C. The amplified products were visualized on 1.8% agarose gel [31, 32]. The capillary sequencer ABI 3730xl (Applied Biosystems, USA) was used to sequence the amplified gene forward and backward. DNA baser 7.0.0 was used to align the forward and reverse sequences to create the contig, and the sequence was then uploaded to NCBI GenBank.

**Table 1:** Primers used in this study

Primer	Sequence 5′ to 3′	Amplicon size (bp)	References
16srRNA (UFF2)	GTTGATCATGGCTCAG	1450 bp	Kumar et al. [33]
16srRNA (URF2)	GGTTCACTTGTTACGACTT		
SWP-1 F	TTGCAGAGTGTTGTTAAGGGTTT	514 bp	Jaroenlak et al. [47]
SWP-1R	CACGATGTGTCTTTGCAATTTTC		
SWP-2 F	TTGGCGGCACAATTCTCAAACA	148 bp	
SWP-2R	GCTGTTTGTCTCCAACTGTATTTGA		
LGBPF	CGGCAACCAGTACGGAGGAAC	115 bp	Used in this study
LGBPR	GTGGAAATCATCGGCGAAGGAG		
PXNF	ATCCAGCAGCCAGGTATG	147 bp	
PXNR	CAGACTCATCAGATCCATTCC		
ProPOF	ACCACTGGCACTGGCACCTCGTCTA	161 bp	
ProPOR	TCGCCAGTTCTCGAGCTTCTGCAC		
Hsp70F	CCTCCTACGTCGCCTTCACAGACA	233 bp	
Hsp70R	GGGGTAGAAGGTCTTCTTGTCTCCC		
Integrin ßF	TTGGGCATCGTGTTCGGACTC	184 bp	
Integrin ßR	TGAAGGTGTTGGTCGCAGGTC		
α2-macroglobulinF	GCACGTAATCAAGATCCG	204 bp	
α2-macroglobulinR	CCCATCTCATTAGCACAAAC		

### Histopathological analysis

The shrimp in the symptomatic and asymptomatic groups were anesthetized with MS222 (Sigma-Aldrich), and tissue samples (hepatopancreas) were collected. The post-mortem examination aimed to identify and document any observable lesions or clinical symptoms in the internal organs of the shrimp. Tissue samples were fixed in Davidson’s solution for 48 h and placed in 70% ethanol solution. After washing, the fixed tissues were sliced into tiny pieces that measured 1–2 mm. The samples were then treated with ethyl alcohol 70%, 80%, 95%, 100%, and xylene solution. Using an impregnation technique, the cleared tissue was embedded in paraffin (Leica EG 1140 H, Germany). Samples were then cut with a microtome to obtain sections with a thickness of 5 μm. Subsequently, the slide samples were stained with Hematoxylin and eosin (H and E), and the infected and healthy shrimp tissues were identified under the microscope at 10X and 40X magnification and labeled for further data analysis. *RNA extraction and Reverse Transcription*.

As directed by the manufacturer, total RNA was extracted through the Trizol^®^ reagent. In summary, 1 mL of Trizol^®^ was used to aseptically homogenise 0.1 g of gut or hepatopancreas tissue samples from the control and symptomatic groups for 15 to 30 s at room temperature. At 20 °C, the homogenate was incubated for five minutes. Subsequently, chloroform (200 µL) was incorporated into the homogenate, vigorously mixed for 15 min at 20 °C, and centrifuged for 10 min at 10,000 rpm. After transferring the upper aqueous layer to a new tube, 500 µL of isopropanol was added. After two hours at -20 °C, the mixture was centrifuged for ten minutes at 10,000 rpm. The pellet was air-dried to remove any remaining ethanol after being cleaned with 75% ethanol and centrifuged for 10 min at 7,000 rpm. The RNA pellets were dissolved in 50 µL of DEPC-treated water and stored at -20 °C until further use. After that, RNase-free DNAse I was used to eliminate any genomic DNA contamination. A spectrophotometer from NanoDrop was used to measure the concentration of RNA (in ng/µL) and check the absorbance at 260/280 (Thermo Scientific, India). Later, the integrity of the RNA was assessed on a 2% agarose gel.

Reverse transcription was then carried out according to the manufacturer’s instructions using the Reverted H Minus First Strand cDNA Synthesis Kit (Thermo Fisher Scientific, India). Briefly, one µg of total RNA and 1 µL of a random hexamer primer solution were combined. 8 µl of the reaction mixture was added, along with 200 units of RevertAidTM H minus M-MuLV reverse transcriptase, 20 units of ribonuclease inhibitor, and 4 µL of 5x reaction buffer (0.25 mol-1 Tris-HCl pH 8.3, 0.25 mol-1 MgCl_2_, 0.05 mol-1 DTT). After five minutes at 25 °C, the reaction mixture was incubated for sixty minutes at 42 °C. The reaction was stopped after five minutes of heating at 70 °C and a cooldown to 4 °C. Samples of cDNA (complementary deoxyribonucleic acid) were examined using PCR and kept at -20 °C for further use. Complementary deoxyribonucleic acid (cDNA) samples were analysed by polymerase chain reaction (PCR) and stored at -20 °C until needed.

### Quantitative real-time PCR (RT-QPCR) analysis

Using StepOnePlus Real-time PCR systems (Applied Biosystems) and a pair of specific primers, the expression of various genes was measured by RT-qPCR [33, 34]. These genes included Peroxinectin (PXN), beta-1, 3-glucan binding protein (LGBP), Prophenoloxidase (ProPO), Heat shock protein 70 (Hsp70), Integrin ß, α2-macroglobulin and β -actin (a house-keeping gene to check for the integrity of RNA) (Table 1). The total volume used for the amplification was 20 µL, with 0.5 µL of each particular primer, 10 µL of 2X Maxima SYBR Green/ROX qPCR Master Mix (Thermo Fisher Scientific), one µL of cDNA (50 ng), and eight µL of nuclease-free water.

The RT-qPCR protocol involved a four-step process to evaluate the expression of target and reference genes. A master mix was prepared in triplicate for each biological replicate. This was followed by 40 amplification and quantification cycles consisting of a sequence of 15 s at 95 °C and 30 s each at 60 °C and 72 °C. A subsequent melting curve analysis was performed between 55 and 95 °C with a heating rate of 0.10 °C per second, accompanied by continuous fluorescence tracking. Post-analysis cooling was conducted at a temperature of 4 °C. In addition, each primer pair included a negative control without the template cDNA to ensure specificity. Gene expression levels were determined by adopting the comparative CT approach, known as the 2-^ΔΔCt^ method, proposed by Livak and Schmittgen (2001), with further validation through Pfaffl’s relative standard curve method from 2002 (Pfaffl, 2002). Statistical relevance was assessed using a t-test on the logarithmically transformed values derived from the formula 2^^ΔΔCT^, with P values below 0.05 indicating significant findings.

### Bacterial enumeration from gut samples of shrimp

The total number of cultivable bacteria was measured in the guts of symptomatic and asymptomatic shrimp samples using a slightly modified version of the standard protocol [35]. In summary, nine shrimp samples from each symptomatic and asymptomatic group were cleaned with 70% alcohol, had their internal organs removed and sliced into tiny pieces, and underwent aseptic dissection. Samples of tissue were homogenized aseptically for 15 to 30 s at room temperature in 10 mL of distilled water. Tryptone soya agar (TSA) media was used to plate aliquots (0.1 mL) of each successive dilution of the homogenate, and the plates were incubated overnight at 28 °C. Next, a physiological saline solution that had been sterilized was used to dilute the homogenate serially. The quantity of bacteria in the fish gut sample was measured, and the number of colonies was counted using plates that contained 30–300 CFU/mL at particular dilutions. A single colony was then selected from TSA plates based on its distinct shape, size, and colour, and it was cultivated overnight at 28 °C with 120 rpm shaking in Tryptone Soya Broth (TSB). The culture was streaked onto TSA medium plates to verify the isolates’ purity (based on colony development). In addition, a single colony was inoculated into a TSB medium and incubated for the whole night at 28 °C with continuous agitation (120 rpm). A stock culture containing 40% glycerol was prepared and stored at -80 °C until needed again.

### Bacterial isolates phylogenetic comparison and identification

Amplification and sequencing of PCR 16 S rRNA amplicons were used to identify bacterial isolates. The 3730xl capillary ABI sequencer (Applied Biosystems, Foster City, CA) was used to sequence the amplified gene products in both forward and reverse directions (Table 1). DNA Baser 7.0.0 was used to align the forward and reverse sequences to assemble the 16 S rRNA sequences. Using BLAST (http://blast.ncbi.nlm.nih.gov), the 16 S rRNA bacterial sequences were assembled, aligned, and compared with existing sequences in the NCBI GenBank database. The sequences were then uploaded to GenBank to create a phylogenetic tree.

The neighbor-joining method and evolutionary analyses were used in MEGA11 [36] to develop recovered bacterial strains’ evolutionary history with the optimal tree [37]. The branch lengths and evolutionary distances, depicted to scale, are used to infer the phylogenetic tree. The analysis was done on 32 nucleotide sequences, and the evolutionary distances were calculated using the Maximum Composite Likelihood method. The units represent the number of base substitutions per site. The final dataset contained 1444 positions overall. Codon positions, e.g., 1st + 2nd + 3rd + non-coding was included. Ambiguous positions were eliminated for each sequence pair using the pairwise deletion option.

### Axenic artemia franciscana cyst hatching and survival assay

High-quality cysts were hatched using the previously outlined decapsulation and hatching processes to create the germ-free brine shrimp *Artemia franciscana* larvae [38]. In brief, 18 mL of distilled water was used to hydrate 200 mg of *A. franciscana* cysts (INVE Aquaculture, Belgium) for one hour. Through decapsulation, sterile cysts and larvae were prepared using 660 µL of 32% NaOH and 10 mL of 50% NaOCl. Aeration with 0.2-µm filtered air was supplied throughout the process. Every instrument used for the manipulation was sterilized and done under a laminar flow hood.

After two minutes, the decapsulation was stopped by adding 10 mL of 10 g/l Na_2_S_2_O_3_. Filtered autoclaved seawater (FASW) with 35 g/L of instant ocean^®^ synthetic sea salt was used to wash the decapsulated cysts. The cysts that had been decapsulated were put back into a 50 mL tube with 30 mL FASW, and they were allowed to hatch for 24 h at 28 °C under continuous illumination of around 27 µE/m^2^ s on a rotor running at 4 rpm. Larvae at developmental stage II (mouth opened to swallow particles) were collected after 28 h of incubation. Spread plating (100 µL) and addition (500 µL) of hatching water to Marine Agar and Marine Broth were used to confirm the axenicity. The incubation period was then five days at 28 °C [39]. Studies that began with larvae that were not axenic were discarded.

Using the axenic brine shrimp larvae, a survival assay was performed to determine the virulence of isolated gut bacteria, as previously reported (Kumar et al. 2018). Brine shrimp larvae that had hatched and reached developmental stage II were collected and volumetrically counted. Twenty larvae in 10 mL FASW in each tube were used for the toxicity test, carried out in sterile 40-mL glass tubes. The gut bacterial isolates were cultured overnight at 28 °C with 120 rpm shaking in TSB. Subsequently, the overnight bacterial suspension was exposed to axenic larvae @ 10^7^ CFU/mL. By counting the number of survivors 48 h after exposure, the pathogenicity of the isolated bacteria was ascertained as previously described [39]. The brine shrimp larvae that were not given the bacterial solution were used as a control group. Five replicates were kept for every treatment group and control group. The Institutional Animal Ethics Committee, ICAR-CIFRI, Kolkata, India, has approved the animal utilization protocol for the experimental setup. The study is reported following the ARRIVE guidelines.

### Statistical analysis

Data were arcsine-transformed to ensure homoscedasticity and normality. Using SPSS version 24.0, the samples were submitted to a one-way analysis of variance (ANOVA) and Duncan’s multiple range test; *P*-values < 0.05 were considered significant.

## Results

### EHP-induced mortality in *L. vannamei*

**Fig. 1:**
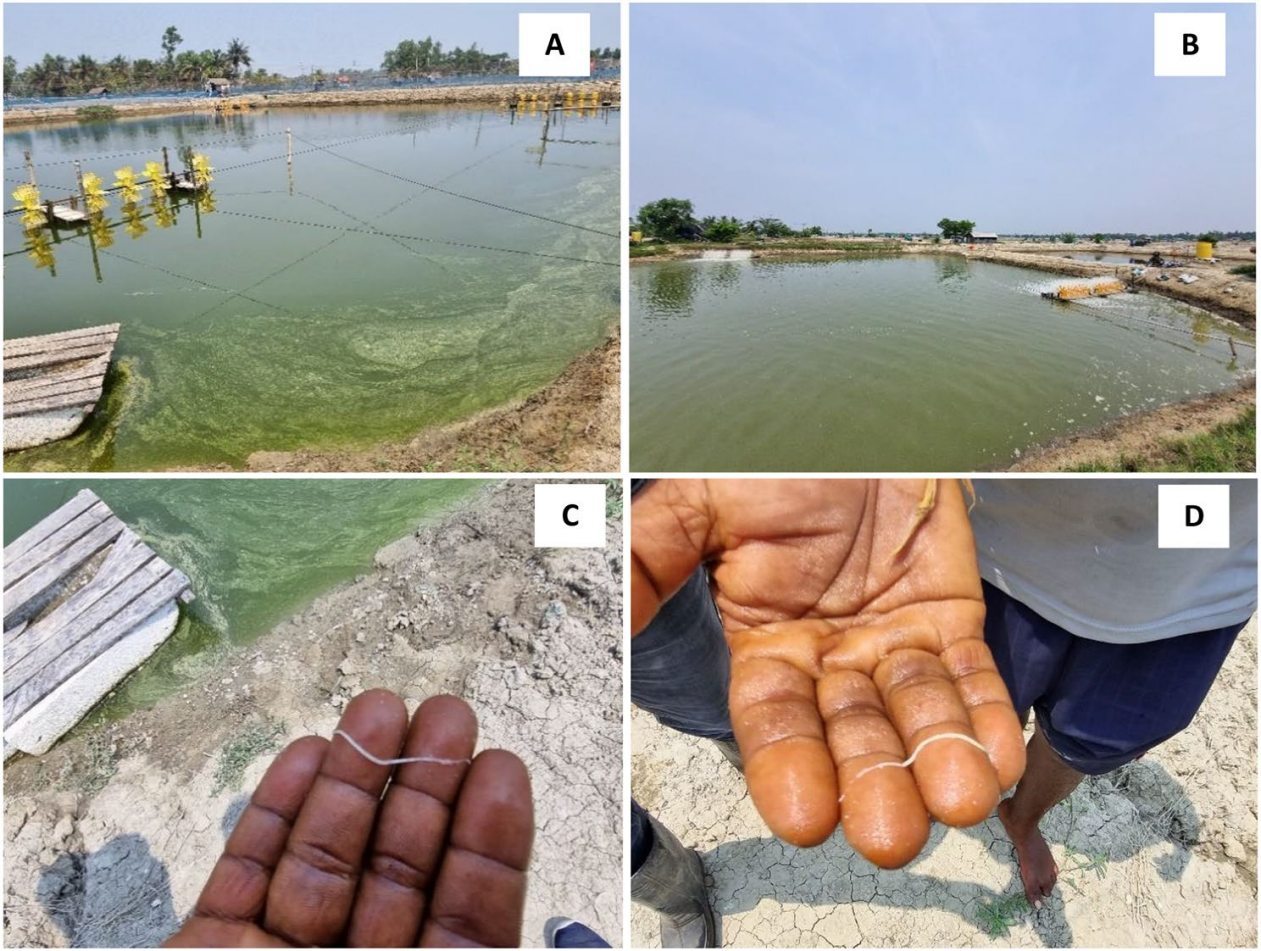
(**A**-**B**) The shrimp farms infected with White Feces Syndrome (WFS) and (**C**-**D**) white feces observed in the cultured water

During sampling, we observed the presence of floating white fecal strings in shrimp ponds. Upon investigation, we found that shrimp exhibited slow growth rate, size disparities (size ranging from 0.5 g to 20 g), softshell appearance, a gastrointestinal region with yellowish to white discolouration, and chronic mortalities (Fig. 1). Passive data collection estimated that about 55% of *L. vannamei* deaths were due to this disease condition. These observations suggest the possible involvement of *Ecytonucleospora hepatopenaei* (EHP) in the shrimp’s poor health and mortality.

**Fig. 2:**
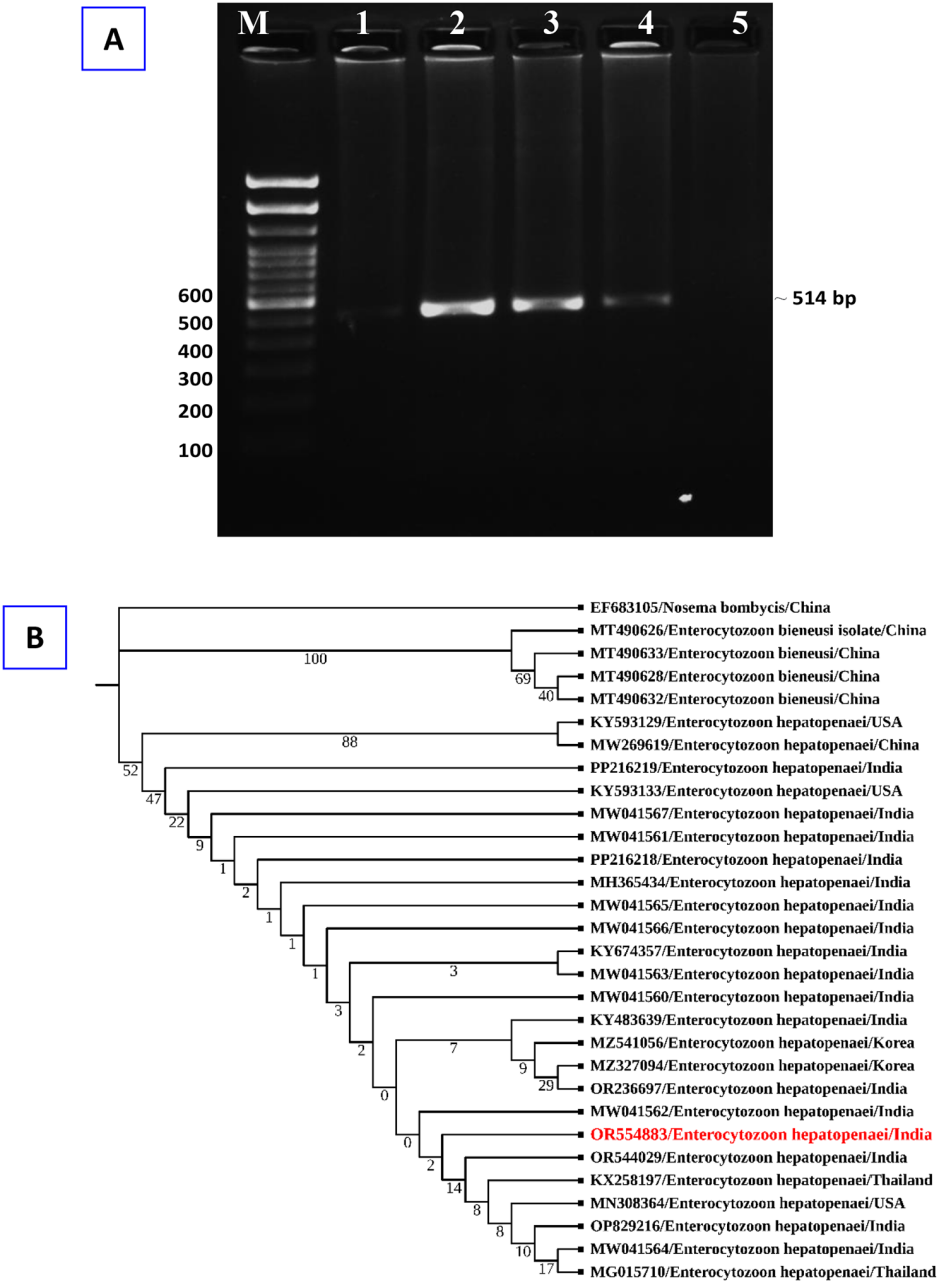
(**A**) Agarose gel of PCR amplicon from shrimp samples using *Ecytonucleospora hepatopenaei* (EHP) specific primers. M − 100 bp DNA ladder. Lane 1 and 5: Exhibits negative amplicons for EHP; Lane 2–4: Positive amplicons (~ 514 bp) for the EHP parasite from template DNA. (**B**) Phylogenetic tree analysis of EHP based on spore wall protein nucleotide sequences following the neighbor-joining method by the MEGA11 software. The numbers next to the branches indicate percentage values for 1000 bootstrap replicates

For EHP detection, the ssu rRNA (small subunit ribosomal RNA) gene of the EHP was amplified using nested PCR utilizing DNA samples taken from the hepatopancreas of the control and symptomatic prawn. PCR assay results exhibited that EHP was present in three hepatopancreas samples, while negative results were observed in two samples examined from shrimp farms during September 2023. Subsequently, the gene sequence analysis confirmed that the isolated microbial species was the microsporidian parasite *Ecytonucleospora hepatopenaei* (EHP) (Fig. 2A). The sequence has been submitted to GenBank with the accession number OR554883. Following a BLAST-N search, the detected sequences were shown to have 100% identity with EHP (NCBI GenBank Accession numbers MG015710, OR544029 and MW041564). A phylogenetic tree was created using the NCBI sequence (Fig. 2B). The extracted DNAs were also examined for other main shrimp viruses (White spot syndrome virus, WSSV; Infectious hypodermal and haematopoietic necrosis virus, IHHNV, and Acute hepatopancreatic necrosis disease, AHPND) to ascertain if the obtained shrimp samples were infected with additional pathogens. WSSV, IHHNV, and AHPND were not found in the collected examined samples.

### Histopathological changes during EHP infection

**Fig. 3:**
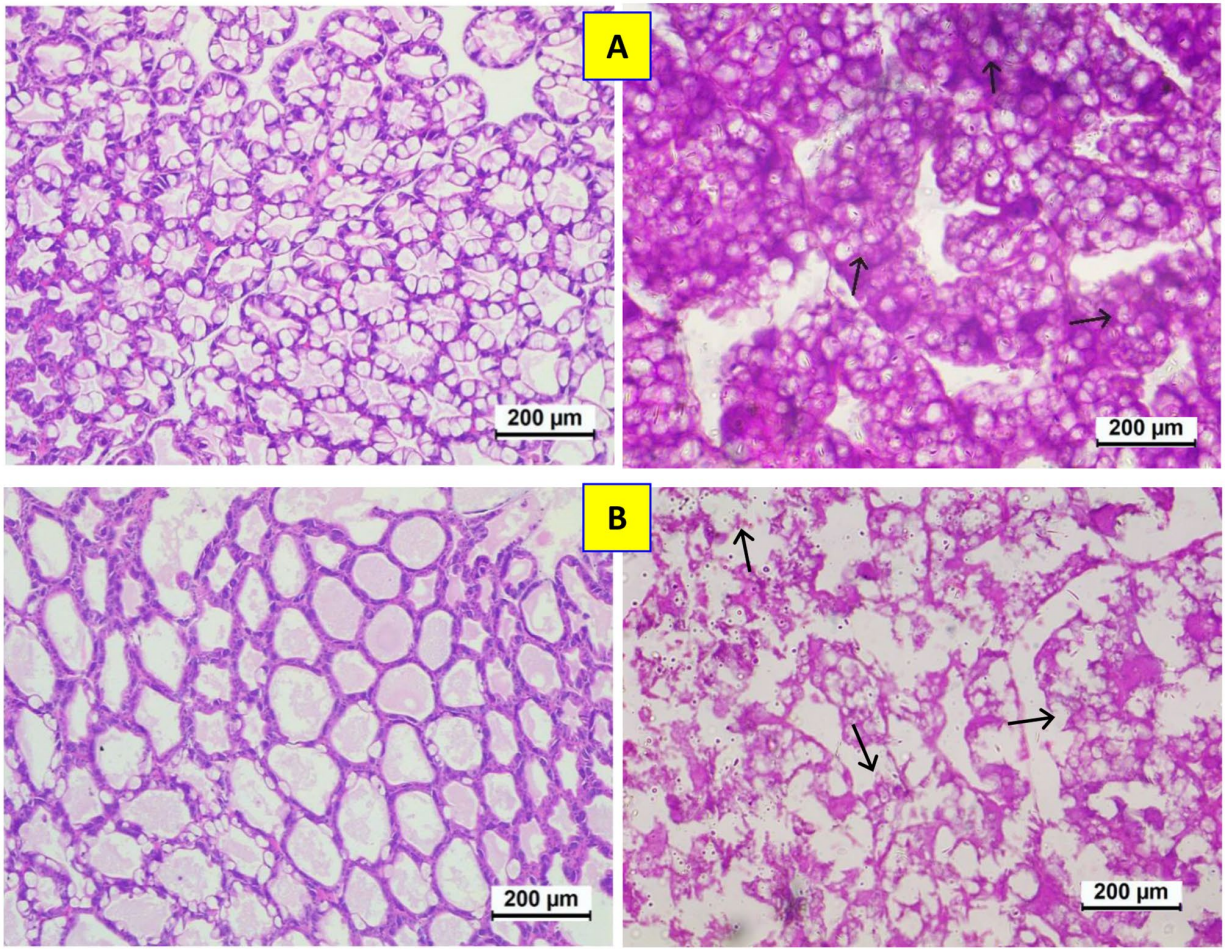
Histopathological investigation of shrimp hepatopancreas. (**A**) Control samples - Normal arrangement of hepatopancreas tubules in healthy hepatopancreas. (**B**) EHP-infected samples - Disordered arrangement of hepatopancreatic tubules and inflammatory cell infiltration in the symptomatic hepatopancreas. Sloughing of hepatopancreas, melanization, and enlargement of the nucleus were also observed

During the investigation of asymptomatic and symptomatic samples (5 shrimp from each group), we found that the control group hepatic tubules were tightly packed, with a star-shaped lumen with a complete structure and an undamaged basement membrane in the healthy hepatopancreas (Fig. 3A). On the other hand, the hepatopancreas tubules were apparent in the cytoplasm of the affected epithelial tubule cells. The hepatopancreas in the symptomatic group showed signs of minor hepatic tubule atrophy together with vacuoles and exfoliated cells between the tubules (Fig. 3B). Prominent vacuoles separated the hepatopancreas’s hepatic tubules, the basement membrane was destroyed, and the cells were exfoliated. Spore clusters were seen in the epithelial cells, the basement membrane was severely disrupted, and the hepatic tubules had more severe damage with a looser shape.

### EHP infection modulates the immune response of *L. vannamei*

**Fig. 4:**
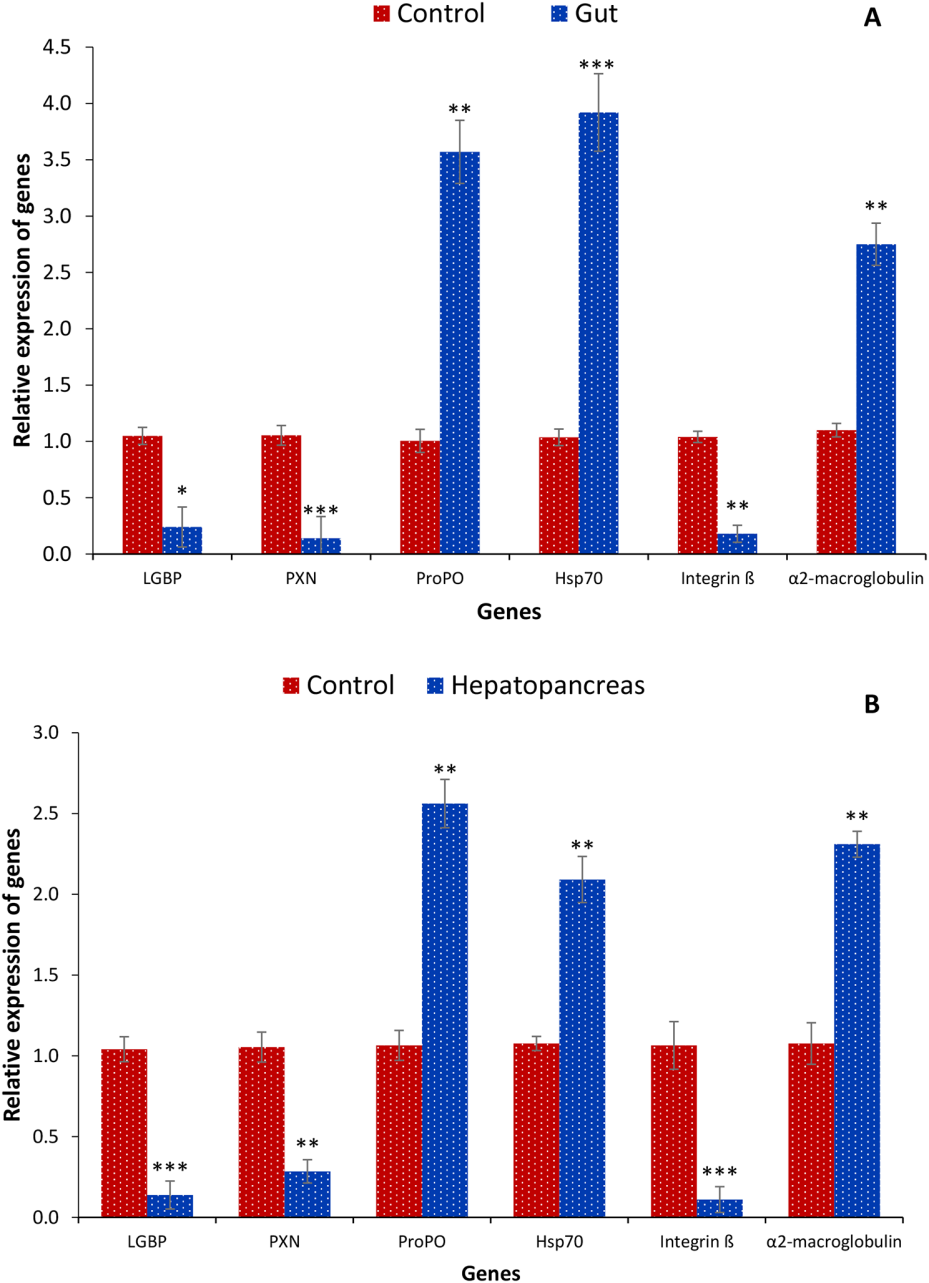
Transcription of immune system-related genes from *symptomatic* and asymptomatic shrimp tissue samples (gut and hepatopancreas). The expression of Lipopolysaccharide and beta-1, 3-glucan binding protein (LGBP), Peroxinectin (PXN), Prophenoloxidase (ProPO), Heat shock protein 70 (Hsp70), Integrin ß, α2-macroglobulin was analyzed in the control and EHP infected group from (**A**) gut and (**B**) hepatopancreas tissue samples. The expression level in the control group was regarded as 1.0, and thereby, the expression ratio of the symptomatic group was expressed in relation to the control group. The results are the mean ± SE (*n* = 3) and the vertical bars with asterisks indicate significant differences between control and EHP infection groups (*P < 0.05, **P < 0.01, ***P < 0.001, ****P < 0.0001, *****P < 0.00001)

EHP infects the primary digestive glands, the hepatopancreas, and the shrimp’s gut. This leads to inadequate nutritional absorption, severe growth retardation or stunted growth, and weakened immunity. Hence, the transcription of pattern recognition receptors and immune system-related genes such as Lipopolysaccharide and beta-1, 3-glucan binding protein (LGBP), Peroxinectin (PXN), Prophenoloxidase (ProPO), Heat shock protein 70 (Hsp70), Integrin ß and α2-macroglobulin was investigated from hepatopancreas and gut samples. The findings highlight that expression of ProPO, Hsp70 and α2-macroglobulin genes were significantly increased in hepatopancreas and gut samples of EHP-infected shrimp compared to control shrimp. In contrast, decreased transcription of LGBP, PXN and Integrin ß was observed in shrimp symptomatic with Hepatopancreatic microsporidiosis (Fig. 4A, B). The results suggest that parasitic infection strongly influences the pro-inflammatory response in shrimp, marked by enhanced transcription of pro-inflammatory cytokine genes.

### Gut microbiota composition of *L. vannamei* during EHP infection

The host’s gut microbiota is crucial in immunomodulation, pathogen defence, gut mucosal barrier preservation, and nutrition metabolism [40]. Growth inhibition and antibacterial action are also considered essential in controlling pathogenic microorganisms [41]. We then used this information to design our sampling method. We hypothesized that a bacterial colony’s distinctive shape, colour, and size would likely indicate a divergent bacterial group. A well-designed culture technique was applied to identify the types of bacteria present in the gut samples of *L. vannamei*. Despite using the established approach, only 14 bacterial strains were isolated from axenic cultures of both symptomatic and asymptomatic gut samples of *L. vannamei* (Table 2).

**Table 2:** Bacterial isolates recovered from control and symptomatic shrimp samples

Sample	Bacterial species	Accession number	Gram staining	Class
Control	*Aeromonas hydrophila*	PP774222	Gram-negative	Aeromonadaceae
	*Citrobacter freundii*	PP774226	Gram-negative	Enterobacteriaceae
	*Enterobacter cloacae*	PP774371	Gram-negative	Enterobacteriaceae
	*Morganella morganii*	PP774229	Gram-negative	Morganellaceae
	*Vibrio campbellii*	PP774383	Gram-negative	Vibrionaceae
	*Vibrio harveyi*	PP774384	Gram-negative	Vibrionaceae
Symptomatic	*Vibrio parahaemolyticus*	PP754438	Gram-negative	Vibrionaceae
	*Vibrio parahaemolyticus*	PP754452	Gram-negative	Vibrionaceae
	*Vibrio harveyi*	PP762096	Gram-negative	Vibrionaceae
	*Vibrio campbellii*	PP762099	Gram-negative	Vibrionaceae
	*Vibrio campbellii*	PP762171	Gram-negative	Vibrionaceae
	*Pseudomonas aeruginosa*	PP762173	Gram-negative	Pseudomonadaceae
	*Enterobacter cloacae*	PP762174	Gram-negative	Enterobacteriaceae
	*Aeromonas hydrophila*	PP762247	Gram-negative	Aeromonadaceae

The isolates’ phylogenetic diversity was evaluated using 16 S rRNA amplicon sequencing data. This method provided preliminary taxonomic clades of the recovered bacterial isolates. The findings indicated that the bacterial isolates were primarily facultative anaerobes that exhibited Gram-negative properties upon Gram staining and belonged to the families Vibrionaceae, Morganellaceae, Enterobacteriaceae, Aeromonadaceae, and Pseudomonadaceae. The bacteria isolates include *Vibrio parahaemolyticus*, *V. harveyi*, *V. campbellii*, *Pseudomonas aeruginosa*, *Enterobacter cloacae*, *Aeromonas hydrophila* and *Citrobacter freundii*. Most isolates were clustered within Vibrionaceae, a bacterial family considered normal microbiota from shrimp; however, few species of this family have been associated with disease outbreaks and mortality. Interestingly, there were also a few species isolates from Enterobacteriaceae, Morganellaceae, Aeromonadaceae and Pseudomonadaceae family from the gut samples of *L. vannamei* (Table 2). Although we could isolate a few bacterial species from gut samples of shrimp, hundreds of non-culturable bacterial species would be present in the shrimp gut, contributing to shrimp health and disease resistance.

**Fig. 5:**
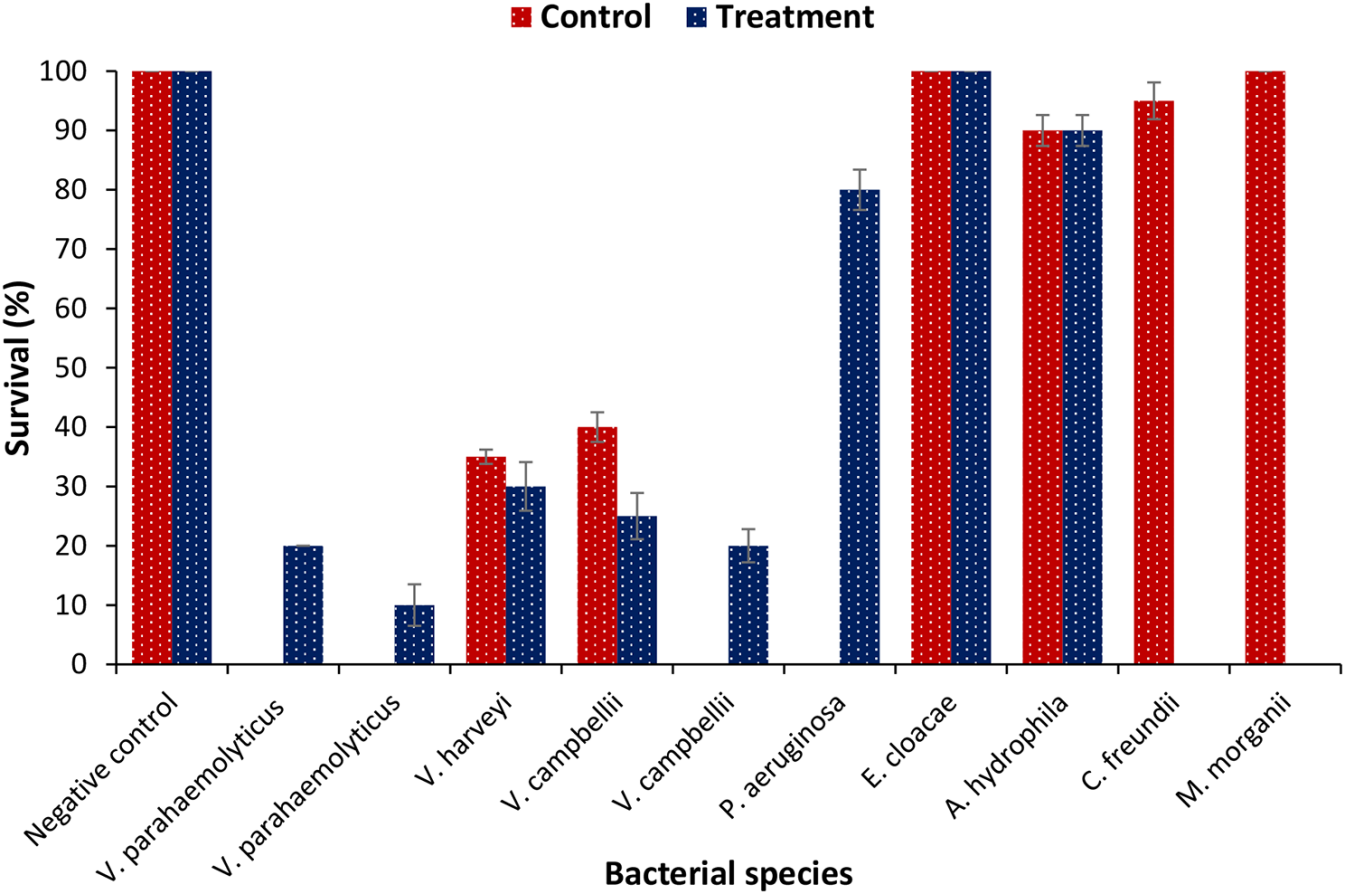
Survival (%) of brine shrimp larvae after 48 h of challenge with different bacterial isolates. The larvae were challenged with bacterial isolates at 10^7^ cells/ml of rearing water. The unchallenged larvae served as control. Error bars represent the standard error of five replicates; different letters indicate significant differences (*P* < 0.05)

To determine the correlation between EHP infection and the pathogenicity of gut bacteria, the in vivo virulence of isolated bacteria was investigated using the crustacean animal model *Artemia franciscana* [42]. Results showed that isolated gut bacteria in control samples were mainly non-pathogenic, except *Vibrio campbellii* and *V. harveyi*, which induced approximately 60% mortality in brine shrimp larvae. In contrast, ~ 80% of gut bacteria isolated from EHP-infected shrimp samples were highly pathogenic, causing 70–80% mortality in brine shrimp larvae within 48 post-challenge (Fig. 5). The results suggest that EHP infection is possibly linked with increased colonization of pathogenic bacteria, resulting in mass mortality during infection.

## Discussion

EHP-caused hepatopancreatic microsporidiosis has been a significant problem for *L. vannamei* cultivation worldwide since the initial discovery of EHP, the shrimp cultivation sector has experienced enormous financial losses due to the difficulty in detecting EHP in the early stages of infection and its rapid vertical and horizontal spread, ultimately resulting in colony-wide infection [43–46]. Moreover, EHP interferes with the energy metabolic process and typically prevents shrimp from growing and developing, resulting in infected shrimp being much smaller in length and weight compared to healthy shrimp. EHP has gained a considerable interest recently; however, most earlier omics research [21, 22] concentrated on collecting samples from shrimp that were naturally infected with EHP. As a result, there is a lack of knowledge on the host immunological response to EHP infection. WFS and coinfection with EHP and other opportunistic *Vibrio* species appear to be connected. Various factors has been proposed as as contributing to the development of WFS, including heavy gregarine infestation, vibriosis and hemocytic enteritis, a particular bacterial taxon known as the “pathobiome,” and varying environmental conditions in a grow-out pond. According to Tangprasittipap et al. [7] and Tang et al. [6], EHP primarily affects the hepatopancreas, the primary digestive gland, impairing nutrient absorption, causing severe growth retardation or stunted growth and low immunity. To better understand the cellular and molecular response mechanism, this study examined the gut microbiota, histopathological alterations, and immune characteristics of shrimp infected with EHP.

The vital digestive organ of crustaceans, the hepatopancreas (HP) carries out the functions of both the liver and the pancreas in shrimp. According to Wang et al. [24], HP is essential for food digestion, absorption, transport, energy reserve storage, and several essential metabolic processes. As a result, the shrimp microsporidian EHP targets HP as its target organ [47, 48]. Histopathological analysis performed during the investigation identified necrotic HP tubules, necrosis, and epithelial cell sloughing. Moreover, the symptomatic hepatopancreas exhibits inflammatory cell infiltration and an unorganized arrangement of hepatopancreatic tubules. Sloughing of hepatopancreas, melanization, and enlargement of the nucleus were also observed. Overall, the analyses suggest that EHP infection induces severe damage to the HP of shrimp.

Like many other invertebrates, crustaceans are an essential branch of animals that primarily rely on their innate immune systems to defend against the invasion of harmful bacteria. Shrimps’ innate immune system consists of humoral and cellular immunological responses work together to provide defense against microbial illnesses [49]. Numerous immunological processes, including phagocytosis and apoptosis *via* immune cells such as hemocytes and the prophenoloxidase system, are involved in the cellular responses. Meanwhile, several non-specific enzymes or other components involved in humoral responses either directly destroy the pathogens or prevent them from growing and spreading [50, 51]. Shrimp’s innate immune system initially uses pattern recognition receptors (PRRs) to identify the pathogens. This allows the immune system to initiate a sequence of downstream immune signaling pathways, triggering humoral and cellular immunological responses [50]. EHP infection has been found to affect the immune response of shrimp and potentially impairing certain functions. In this study, EHP infection modulates the transcription of innate pattern recognition receptors and immune system-related genes such as Lipopolysaccharide and beta-1, 3-glucan binding protein (LGBP), Peroxinectin (PXN), Prophenoloxidase (ProPO), Heat shock protein 70 (Hsp70), Integrin ß and α2-macroglobulin. Upregulation of ProPO, Hsp70 and α2- macroglobulin genes were observed in EHP-infected shrimp compared to control shrimp. In contrast, decreased transcription of LGBP, PXN and Integrin ß was observed in shrimp infected with hepatopancreatic microsporidiosis. These observations suggest that EHP infection may have activated or suppressed different aspects of the immune system, affecting the growth and survival of *L. vannamei* in the aquaculture system.

Though research on the impact of EHP infection on the physiology of shrimp has recently advanced, little information is available on the structure and function of the gut microbiota [19]. Nowadays, it is well acknowledged that the gut microbiota plays essential roles in immunoregulation, pathogen invasion prevention, and host nutrition acquisition [26, 27]. On the other hand, pathogenic incursions can significantly disturb the gut microbiota’s assembly [27, 29]. Infections and co-infections in the taxonomically varied gastrointestinal (GI) tract result from colonization by an alternative microbe that upsets the intestinal microbiota [52, 53]. Furthermore, it’s becoming more evident that gut symbionts play critical roles in nutrition absorption and forming barriers against pathogen invasion [20, 54]. While little is known about the precise immunological mechanisms that the gut microbiota of shrimp provides, there is evidence that these mechanisms interact with digestive processes to directly impacting the growth of shrimp and the severity of diseases [55]. Therefore, this study investigated how and to what extent the EHP infection modified the composition of shrimp gut microbiota, and its impact on the immune response. Our findings demonstrate that EHP modulates the shrimp hepatopancreas, causing growth inhibition, size disparity, loss of appetite, and chronic mortality. The gut microbiota study further substantiates that the presence of pathogenic bacteria, e.g., *Vibrio parahaemolyticus*, *V. harveyi* and *V. campbellii* likely has synergistic actions with the microsporidia EHP, increasing the severity of the infection, making shrimp more vulnerable to pathogen invasion.

In conclusion, this is the first documented study that provides baseline information on the *Ecytonucleospora hepatopenaei* prevalence, causing growth retardation and severe size variation in *Litopenaeus vannamei* from Eastern India shrimp farms. The results showed that EHP infection regulates gut microbial and cellular histopathological profiles, which are closely related to differential immune-related gene expression and ultimately modulate the immune system and the survival of shrimp. The EHP-infected shrimp group has an increased relative abundance of *Vibrio parahaemolyticus*, *V. harveyi*, and *V. campbellii*, suggesting that EHP infection promotes the increase of pathogenic bacteria of pathogenic bacteria. Therefore, to protect shrimp farms, it is important to encourage producers to consider standard management strategies, such as regular monitoring and the use of EHP-free shrimp for stocking, lowering the shrimp densities, and maintaining good water and sediment quality. Nevertheless, these findings could aid in developing immunoprophylactic strategies to enhance the innate or nonspecific immunity of *L. vannamei* and combat EHP infection.

## Data Availability

Isolated parasite (EHP) accession numbers: OR554883. Isolated gut bacteria accession numbers: PP774222, PP774226, PP774371, PP774229, PP774383, PP774384, PP754438, PP754452, PP762096, PP762099, PP762171, PP762173, PP762174, PP762247.
